# Alkaloids and Selected Topics in Their Thermochemistry

**DOI:** 10.3390/molecules26216715

**Published:** 2021-11-06

**Authors:** Maja Ponikvar-Svet, Diana N. Zeiger, Joel F. Liebman

**Affiliations:** 1 Department of Inorganic Chemistry and Technology, Jožef Stefan Institute, Jamova 39, SI-1000 Ljubljana, Slovenia; 2 Department of Chemistry and Biochemistry, University of Maryland, Baltimore County, Baltimore, MD 21250, USA; dzeige1@umbc.edu

**Keywords:** alkaloids, (de)methoxylation, condensed phase, enthalpy of formation, thermochemistry

## Abstract

Alkaloid chemistry is varied and complex. Many alkaloids attract a great deal of interest because of their physiological activity, yet surprisingly little is known about the thermochemistry of these compounds, especially in the gas phase. In this paper, we investigate the thermochemical characteristics, specifically demethoxylation enthalpies rather than those derived from trans-methoxylation reactions, of a series of biologically relevant alkaloids in their condensed phase.

## 1. Introduction

Let us start with the definition of an alkaloid. In the public/popular literature, alkaloids are defined as “any of numerous usually colorless, complex, and bitter organic bases (such as morphine or caffeine) containing nitrogen and usually oxygen that occur especially in seed plants and are typically physiologically active” [1]. In an official scientific source [2], we find the following: “Basic nitrogen compounds (mostly heterocyclic) occurring mostly in the plant kingdom (but not excluding those of animal origin). Amino acids, peptides, proteins, nucleosides and nucleotides, nucleic acids, amino sugars and antibiotics are not normally regarded as alkaloids. By extension, certain neutral compounds biogenetically related to basic alkaloids are included”. Alkaloids have been extensively studied by the chemical community. A simple SciFinder search [3] results in over 100,000 references to the word “alkaloid” as entered and double that number “containing the concept ‘alkaloid’”. Many studies of individual species do not mention “alkaloid” at all. Given this high level of intellectual activity, it is therefore necessary to consider only selected topics of alkaloid chemistry in our review.

Most simply and most generally, thermochemistry may be understood to interrelate the structure and energetics of chemical species. Again, brevity is demanded. We will limit our attention to the quantity “enthalpy of formation”, that is to say, the amount of heat evolved when a species of interest is formally synthesized from the elements in their standard state (carbon as solid graphite; hydrogen, nitrogen, and oxygen as diatomic gases, all at 25 °C and one atm). This quantity is almost never directly measured but is derived from the experimentally realizable enthalpy of combustion, which essentially is the heat evolved when the species of interest is reacted with oxygen to form gaseous carbon dioxide, liquid water and gaseous nitrogen. Our personal preference has generally been to consider all species of interest in the gaseous state, which obviates the consideration of all intermolecular interactions. A few alkaloids are found as liquids, but most are generally solids. However, experimental studies of the energetics of virtually no alkaloids have ever been undertaken in the gas phase, given that the necessary measurements of enthalpy of vaporization or sublimation remain unreported. This is perhaps not surprising because most alkaloids are multiply substituted, impressively high molecular weight species that have high melting and boiling points. These phase transitions are no doubt often accompanied by decomposition and so fusion and vaporization enthalpies are not particularly useful. By contrast, some/many of the underlying scaffolds (e.g., pyrrolidine, pyridine, quinoline, isoquinoline) are more stable and more volatile. As such, their thermochemistry (in both the condensed and gas phase) is more accessible and more meaningful. We accept this lack of knowledge about the enthalpy of formation values related to the gas phase but instead ask about the “plausibility” of the condensed-phase literature numbers. Thus, contemporary analysis of the few thermochemically characterized alkaloids (Table 1), all in their condensed phase (nearly all solid), is the subject of the present study. The compounds discussed include compounds involved in (1) trans-methoxylation processes involving diastereomers (quinine and related quinidine and the resulting cinchonidine and related cinchonine; (2) demethoxylation of brucine to strychnine and of anisine to amarine; (3) demethoxylation involving papaverine and 1-benzylisoquinoline.



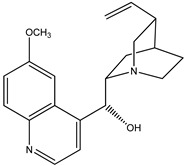



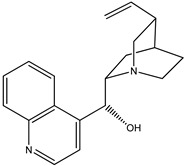

(**1**) quinine, C_20_H_24_N_2_O_2_(**2**) cinchonidine, C_19_H_22_N_2_O

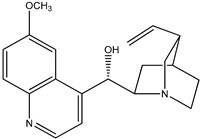



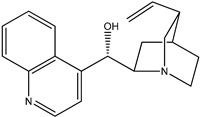

(**3**) quinidine, C_20_H_24_N_2_O_2_(**4**) cinchonine, C_19_H_22_N_2_O

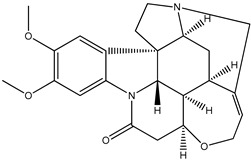



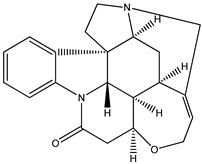

(**5**) brucine, C_23_H_26_N_2_O_4_(**6**) strychnine, C_21_H_22_N_2_O_2_

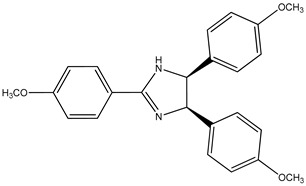



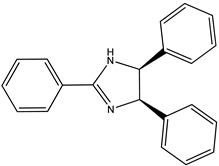

(**7**) anisine, C_24_H_24_N_2_O_3_(**8**) amarine, C_21_H_18_N_2_

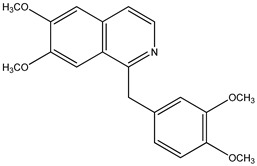



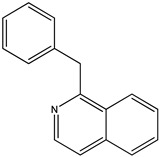

(**9**) papaverine, C_20_H_21_NO_4_(**10**) 1-benzylisoquinoline, C_16_H_13_N

Paralleling those of the alkaloids of interest, we now give the names, formulas and structures of the unsubstituted nitrogen-containing heterocycles whose derivatives are presented in our text.



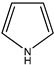



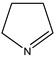



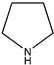



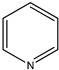

(**11**) pyrrole, C_4_H_5_N(**12**) 1-pyrroline, C_4_H_7_N(**13**) pyrrolidine, C_4_H_9_N(**14**) pyridine, C_5_H_5_N

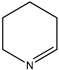



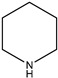



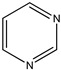



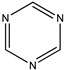

(**15**) 1-piperideine, C_5_H_9_N(**16**) piperidine, C_5_H_11_N(**17**) pyrimidine, C_4_H_4_N_2_(**18**) 1,3,5-triazine, C_3_H_3_N_3_


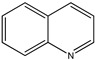



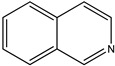



(**19**) quinoline, C_9_H_7_N(**20**) isoquinoline, C_9_H_7_N


## 2. Discussion of Alkaloids from a Thermochemical Perspective

We acknowledge that there have been few literature studies of the enthalpy of combustion, and the derived enthalpies of formation, of any alkaloid at all (all to be discussed below, but mostly taken from the almost 50-year-old thermochemical compendium [4]. The original sources are generally 70 years older than this source.) Perhaps surprisingly, and admittedly disappointingly, there are few theoretical (i.e., non-experimental) estimates of the latter quantity [5,6,7]. These papers result in discrepancies of but a few percent between theory and experiment in the values for enthalpy of combustion, generally 50–100 kJ mol^−1^. Should the reader be content, if not pleased, by such accord? In fact, we are not content because the measurement uncertainties in enthalpies of combustion are necessarily less than those in enthalpies of formation (the enthalpies of formation of combustion products from compounds with C, H, N, and O are insignificant in the present context). To exacerbate the situation, few of the relevant measured quantities are accompanied by any uncertainties or error bars of their own: we are sure they are large when compared to those of most contemporary calorimetric measurements of C-, H-, N-, and O-containing species, typically a “few” kJ mol^−1^. With admitted hesitation, we will consider experimentally determined enthalpies of formation of the alkaloids of interest rather than any of the calculated values. For reference, all values will be given to the nearest integer value in kJ mol^−1^.

There are disappointingly few contemporary determinations of the enthalpy of formation of any alkaloid. These new measurements of alkaloid enthalpies of formation include those of berberine chloride dihydrate (**21**) (often, but erroneously, named the hydrochloride salt) [8] and nicotine (**22**) [9]. These species have the following systematic names: 5,6-dihydro-9,10-dimethoxy-benzo[*g*]-1,3-benzodioxolo[5,6-*a*]quinolizinium chloride dihydrate and 3-[(2*S*)-1-methyl-2-pyrrolidinyl]pyridine, respectively. In the present paper, we will generally use the trivial names for compounds of interest.

We note that in this paper, we will not discuss berberine chloride dihydrate at all. It alone, of all of the species discussed herein, is recognized to be an ionic salt. Therefore, by definition, any comparisons we wish to make between this species and the many neutral nitrogen-containing heterocycles are beyond the scope of the present study.

Domalski (our archival source, [4]) reported the enthalpy of formation of liquid nicotine to be 39 kJ mol^−1^ (the original source cited therein are [10]) but is 68 kJ mol^−1^ according to the contemporary measurements [9]). The reason for the discrepancy is not apparent; we wonder about the reasonably ignored presence of nicotine trihydrate or of any other plausible hydrate [11].

We note that none of these thermochemical studies on nicotine [9,10] compared the simultaneously calorimetrically and calculational determined enthalpies of formation for either of the following species:

(a) The de-methylated species recognized as the alkaloid “nor-nicotine,” with the systematic name 3-(2*S*)-2-pyrrolidinyl-pyridine.
(b) The nicotine isomer in which the methylpyrrolidine is replaced by piperidine to form the alkaloid “anabasine (**23**),” with the systematic name 3-(2*S*)-2-piperidinyl-pyridine.

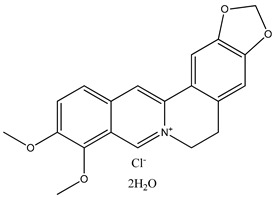



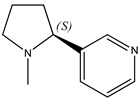



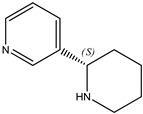

(**21**) berberine chloride dihydrate, C_20_H_18_NO_4_∙Cl∙2H_2_O(**22**) nicotine, C_10_H_14_N_2_(**23**) anabasine, C_10_H_14_N_2_

It appears that neither nor-nicotine nor anabasine has attracted the attention of the thermochemical community. Indeed, nicotine dominates the general discussion of tobacco-derived alkaloids and related synthetic heterocycles. A SciFinder search [3] uncovered more than 100,000 references to nicotine, but only ~2000 and ~2700 references to nor-nicotine and anabasine, respectively).

We recognize alkaloids as generally multifunctional, very often involatile nitrogen heterocycles; nevertheless, we wish to provide thermochemical comparisons involving these species. We ask, however implicitly, how thermoneutral are the following two gas-phase transmethylation reactions R1 and R2 (mass balanced reactions presented below):

nicotine + pyrrolidine → nor-nicotine + *N*-methylpyrrolidine (R1)

(C_10_H_14_N_2_+ C_4_H_9_N → C_9_H_12_N_2_ + C_5_H_11_N)

nicotine + piperidine → anabasine + *N*-methylpyrrolidine (R2)

C_10_H_14_N_2_+ C_5_H_11_N → C_10_H_14_N_2_ + C_5_H_11_N

Likewise, we may consider these reactions in the condensed phases of liquid and/or solid. We note that nicotine and anabasine are isomers, as are piperidine and *N*-methylpyrrolidine, and we naturally ask about the energetics of reactions R3 and R4:

nicotine → anabasine(R3)

*N*-methylpyrrolidine → piperidine (R4)

(In our study, we tacitly assume that reactions R3 and R4 have the same exothermicity, ~30 kJ mol^−1^ for species in the gas phase and ~40 kJ mol^−1^ for species in the liquid phase [12]; the 10 kJ mol^−1^ phase-dependent difference reflects the N∙∙∙H∙∙∙N hydrogen bond in condensed-phase piperidine.)

Admitting that gas-phase values of enthalpies of formation for alkaloids are all but absent, we observe that there are but a few alkaloids for which we have determinations of their condensed-phase thermochemical properties. How can we study any reactions analogous to reactions R1 and R2 for condensed-phase alkaloids, and do we find thermoneutrality? An analysis of the few thermochemically characterized alkaloids, all in their condensed phase (nearly all solid), is the theme of the present study.

Before we proceed, let us emphasize our use of condensed phase thermochemical data is unavoidable. In other words, we now briefly address why are the desired phase change enthalpies (fusion, vaporization and sublimation) absent? We might have expected these data to appear in perhaps archival sources and referenced [13,14]. Certainly, we find available, reliable data for alkaloid building blocks, most notably simply-substituted derivatives of pyrrole, pyridine, quinoline, isoquinoline and their hydrogenated derivatives. (See structures below these far simpler species.) In most cases, diverse thermochemical investigations continue because all of these species are much more volatile and more heat stable. Related volatility and enhanced heat stability are also found as subjects for the blossoming study of natural polycyclic hydrocarbons and nonpolar oxygen-containing derivatives. These species often have structural complexity and “poetic” names that parallel those of alkaloids (e.g., [15]). Summarizing, however frustrating this may be, contemporary studies of alkaloids of any type are invariably for condensed phase species.

The first reaction R5 (mass balanced reaction presented below) we will consider is a trans-methoxylation process involving diastereomers, where we remember that quinine (**1**) and quinidine (**3**) are so related, and cinchonidine (**2**) and cinchonine (**4**) are so related. (Almost perversely in terms of their names, quin*ine* and cinchonidine have the same stereochemistry, as do quinidine and cinchon*ine*.) The systematic names for these alkaloids are lengthy; generally, semi-systematic names are more “officially” used. In other words, the root name cinchonane is used for the framework-related species, namely, the compound that lacks all “methoxy” and “ol” substituents.

(a) Quinine, 6′-methoxy-cinchonan-9-ol, (8α,9R)-, (*R*)-[(2*S*,4*S*,5*R*)-5-ethenyl-1-azabicyclo[2.2.2]octan-2-yl]6-methoxyquinolin-4-yl)methanol
(b) Cinchonidine, cinchonan-9-ol, (8α,9*S*)-, (*R*)-[(2*S*,4*S*,5*R*)-5-ethenyl-1-azabicyclo[2.2.2]octan-2-yl](quinolin-4-yl)methanol
(c) Quinidine, 6′-methoxy-cinchonan-9-ol, (9*S*)-, (2-Ethenyl-4-azabicyclo[2.2.2]oct-5-yl)-(6-methoxyquinolin-4-yl)-methanol
(d) Cinchonine, cinchonan-9-ol, (8α,9*R*))-, (*S*)-[(2*R*,4*S*,5*R*)-5-ethenyl-1-azabicyclo[2.2.2]octan-2-yl](quinolin-4-yl)methanol

quinine + cinchonine → cinchonidine + quinidine(R5)

C_20_H_24_N_2_O_2_ + C_19_H_22_N_2_O → C_19_H_22_N_2_O + C_20_H_24_N_2_O_2_

As written, using the literature values [4] for the enthalpies of formation of the four condensed phase alkaloids this formal solid-phase reaction is exothermic by approximately 4 kJ mol^−1^. We note that the enthalpy of formation lacks error bars for the various quantities; therefore, we must temper our enthusiasm. Moreover, the entirely plausible near-equality of the enthalpies of formation of the pair of diastereomers quinine and quinidine, and of those of the likewise diastereomeric cinchonine and cinchonidine, would necessarily result in this near-zero, or perhaps we should say quite small, enthalpy of reaction. (We must be honest: enantiomers necessarily have identical enthalpies of formation. We do not know of any rule or quantitation method that speaks to the non-zero difference for a pair of diastereomers.) As an example of small differences of formation found for diastereomers we cite the ca. 15 kJ mol^−1^ difference that span the contemporary measurements of the enthalpies of combustion and hence of formation, of the highly functionalized five-carbon aldose sugars: arabinose, lyxose, ribose and xylose, the diastereomeric 2,3,4,5-tetrahydroxypentanals—or more properly, their isomeric pyranoses, the cyclic 1,2,3,4-tetrahydroxylated tetrahydropyrans [16]. By contrast, the isomeric polyether 1,3,5,7,9-pentaoxacyclodecane is calorimetrically shown to be less stable than these sugars by almost 200 kJ mol^−1^ [17].

In the present study, we wish to explicitly consider demethoxylation enthalpies [18] rather than those derived from trans-methoxylation reactions. Now, how reasonable are the derived ~190 kJ mol^−1^ differences between the measured enthalpies of formation of quinine and its demethoxylated counterpart cinchonidine, and between those of the so-related quinidine and cinchonine? To that end, consider the enthalpies of formation of other methoxy species and their corresponding demethoxylated counterparts, where all thermochemical data refer implicitly to the solid-state. Said differently, we are discussing the quantity of “demethoxylation enthalpies”, the difference of the enthalpies of formation for corresponding pairs of molecules ROCH_3_ and RH rather than the enthalpies of the balanced trans-methoxylation reactions R6

ROCH_3_ + R_ref_H → RH + R_ref_OCH_3_ (R6)

where R_ref_OCH_3_ and R_ref_H are some other pair of species. As long as R_ref_ is taken to be the same for all of the comparisons, then the difference of the trans-methoxylation enthalpies of reactions R6 and R7

R’OCH_3_ + R_ref_H → R’H + R_ref_OCH_3_ (R7)

is numerically the same as that of the difference of our demethoxylation enthalpies connecting the enthalpies of formation of ROCH_3_ and RH with those of R’OCH_3_ and R’H.

### 2.1. The Enthalpies of Formation of Brucine and Strychnine

We start with the enthalpy of formation data for some alkaloids. We recognize brucine (**5**) as a dimethoxylated derivative of the much-better-known alkaloid strychnine (**6**); the semisystematic names of these species are 2,3-dimethoxy-strychnidin-10-one and strychnidin-10-one, respectively.

According to the early experimental literature [4], we find the corresponding enthalpies of formation, as solids are −172 kJ mol^−1^ and −496 kJ mol^−1^, respectively. The difference is approximately 324 kJ mol^−1^, or 162 kJ mol^−1^ per methoxy group. We consider this value to be not particularly dissonant from that found for quinine and its counterparts (i.e., 190 kJ mol^−1^).

We note that the two methoxy groups in brucine are located in the ortho position, which is known to be destabilizing; more precisely, *o*-dimethoxybenzene is less stable than either its *m*- or *p*-isomer [19]. Admittedly, this is for the gas phase, given that there are gas-phase enthalpy of formation data for the *o*- and *m*- species but not for *p*-, and there are such data for the *p*-species alone as a solid. Therefore, let us use the measured value for the enthalpy of formation of solid *p*-dimethoxybenzene: −296 kJ mol^−1^ [19,20]. From the recommended, evaluated value found for the enthalpy of formation of gaseous benzene and the enthalpy of sublimation of solid benzene [21], we deduce an enthalpy of formation for solid benzene of 38 kJ mol^−1^. The difference between the enthalpies of formation of *p*-dimethoxybenzene and benzene itself is thus 334 kJ mol^−1^, corresponding to a demethoxylation enthalpy per methoxy group of 167 kJ mol^−1^, encouragingly close to that found for the difference between brucine and strychnine.

Going through [4], we find yet another pair of are the alkaloids: anisine (**7**) and amarine (**8**), which have the systematic names 4,5-dihydro-2,4,5-tris(4-methoxyphenyl)-1*H*-imidazole, (4*R*,5*S*)-*rel*-.4,5-dihydro-2,4,5-triphenyl-1*H*-imidazole (4*R*,5*S*)-*rel*-, and 4,5-dihydro-2,4,5-triphenyl)-1*H*-imidazole, (4*R*,5*S*)-*rel*-.

### 2.2. The Enthalpies of Formation of Anisine and Amarine

The respective measured enthalpies of formation [4] of anisine and amarine, as solids are –264 kJ mol^−1^ and 213 kJ mol^−1^, which corresponds to a difference of 477 kJ mol^−1^. There are three methoxy groups in the latter species, one apiece on each phenyl group; thus, a demethoxylation enthalpy of 159 kJ mol^−1^ per methoxy group is found. This value is consistent with the values we observed previously.

We note now that there is another, albeit totally unrelated, compound that also has the name “amarine”. Its formula and proper name are C_32_H_46_O_8_ and 25-(acetyloxy)-2,16,20-trihydroxy-9-methyl-19-norlanosta-5,23-diene-3,11,22-trione, (2β,9β,10α,16α,23*E*)- (**24**), respectively. We know of no logical, not even etymological, connection between these two identically named “amarine” species; thus, this is a clear example of how care must always be taken in discussion of natural products and their trivial, poetic, nondescriptive, and indeed scientifically meaningless names.



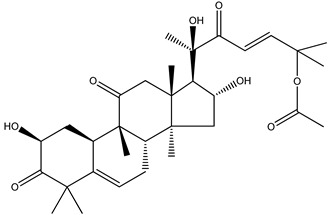

(**24**) “amarine”, C_32_H_46_O_8_

### 2.3. The Enthalpies of Formation of Papaverine and Related Isoquinolines and Isoquinolines

We now turn to papaverine (**9**). In reference [4], we find the desired thermochemical data for papaverine (systematic name 1-[(3,4-dimethoxyphenyl)methyl]-6,7-dimethoxyisoquinoline) with its solid-phase enthalpy of formation of –503 kJ mol^−1^. What is its demethoxylation enthalpy? There are no thermochemical data for the desired species, 1-(phenylmethyl)isoquinoline, nor for that matter are there such data for any alkylated isoquinoline. Indeed, the only simply substituted isoquinoline derivative with an accompanying measured enthalpy of formation is the 5-hydroxy species [22], also see [23].

Regarding the isomeric quinoline derivatives, we note that the enthalpies of formation of the liquid-phase isomeric 2-, 4-, and 6-methylquinolines are nearly the same, ~4 kJ mol^−1^ [24], and those of the isomeric solid 2,6- and 2,7-dimethylquinolines are again nearly the same, ~35 kJ mol^−1^ [25]. The enthalpy of formation is known for 2-methylquinoline (13 kJ mol^−1^) [26], but not for any of the other methylated species. The enthalpy of formation of solid 2-methylquinoline is thus 80 kJ mol^−1^. This seeming indifference to the location of the methyl groups suggests the near thermoneutrality of the following reaction R8 (mass balanced reaction presented below):

2-methylquinoline + isoquinoline → quinoline + 1-methylisoquinoline(R8)

C_10_H_9_N + C_9_H_7_N → C_9_H_7_N + C_10_H_9_N

The near equality of the measured enthalpies of formation of quinoline and isoquinoline [27] thus suggests that the enthalpies of formation of solid 2-methylquinoline and 1-methylisoquinoline should be nearly equal as well. Similarly, we would assume near thermoneutrality for the following reaction R9 (mass balanced reaction presented below):

1-methylisoquinoline + diphenylmethane → 1-benzylisoquinoline + toluene(R9)

C_10_H_9_N + C_13_H_12_ → C_16_H_13_N + C_7_H_8_

Using reported values for the enthalpies of formation of solid diphenylmethane and toluene [21], we deduce that solid 1-benzylisoquinoline (**10**) has an enthalpy of formation of 150 kJ mol^−1^. The demethoxylation enthalpy of papaverine is thus 655 kJ mol^−1^; because there are four methoxy groups, the desired value of 164 kJ mol^−1^ per methoxy group is encouragingly in accord with those of brucine (to form strychnine) and anisine (to form amarine).

What about the demethoxylation enthalpies of other, but admittedly not alkaloid-related, heterocyclic species? Let us use contemporary calorimetric studies to give us the desired solid-phase enthalpies of formation. One such pair of non-alkaloidal species is 2,4-dimethoxypyrimidine and its parent heterocycle, pyrimidine. The enthalpy of formation of the former is −277 kJ mol^−1^ [28], and the latter value, as recently reevaluated, is 133 kJ mol^−1^ [29], resulting in a difference of 410 kJ mol^−1^ or 205 kJ mol^−1^ per methoxy group.

Yet another such pair is 2,4,6-trimethoxy-1,3,5-triazine (trimethyl cyanurate) and 1,3,5-triazine; the enthalpy of formation of the former species is −479 kJ mol^−1^ [30,31], and that of the latter is 172 kJ mol^−1^ [32]. The desired demethoxylation enthalpy is thus 651 kJ mol^−1^, or 217 kJ mol^−1^ per methoxy group.

What can we learn from measurements for liquid-phase species? In the literature, we find the enthalpy of formation values for 2- and 4-methoxypyridine [18] of −88 and −76 kJ mol^−1^, respectively. From [33,34] we find a consensus value of 100 kJ mol^−1^ for the enthalpy of formation of pyridine. The liquid-phase demethoxylation enthalpy of the methoxypyridines is 188 kJ mol^−1^ for the 2-isomer and 176 kJ mol^−1^ for the 4-isomer. Relatedly, the enthalpy of formation of 2,6-dimethoxypyridine is −287 kJ mol^−1^ [18], and so its demethoxylation enthalpy is ~193 kJ mol^−1^ per methoxy group. We know of no such corresponding thermochemical data for the isomeric 3-methoxypyridine, nor for any of the five other isomeric dimethoxypyridines.

Another pair is the liquid-phase species 6-methoxyquinoline and its parent heterocycle, quinoline. The enthalpy of formation of the former is −26 kJ mol^−1^ [35], and that of the latter is 141 kJ mol^−1^ [27]. The demethoxylation enthalpy of 6-methoxyquinoline is 167 kJ mol^−1^. We know of no thermochemical data for any of the other isomeric methoxyquinolines. Why is this value so different from that of 2- and 4-methoxypyridine?

### 2.4. Thermochemical Considerations of Imines, Imidates and Related Species

Next, we consider aliphatic and alicyclic demethoxylation examples involving imines and imidates. The enthalpy of formation of the acyclic species liquid *N*,*O*-dimethyl acetimidate (−204 kJ mol^−1^) may be deduced by summing the enthalpy of formation of liquid *N*,*N*-dimethylacetamide, −278 kJ mol^−1^ [36], and the liquid-phase enthalpy of isomerization interrelating these isomers, 74 kJ mol^−1^ [37].

The imidate has the (*E*)-structure [38], and so the demethoxylation analysis makes use of the (Z)-imine alternatively known as both *N*-methylacetaldimine and 2-azabutene. We know of no experimental measurements for the enthalpy of formation of this imine. From quantum chemical calculations [39,40] we find a consensus value of 54 kJ mol^−1^ for the enthalpy of formation. This theoretical value is for the gas-phase species. Let us approximate the necessary enthalpy of liquefaction (i.e., the enthalpy of vaporization with the opposite sign) as that of pyridine with a total of three carbons [41], resulting in a value of 29 kJ mol^−1^. Accordingly, the enthalpy of formation of the liquid imine is 25 kJ mol^−1^. The demethoxylation enthalpy of *N*,*O*-dimethyl acetimidate (3-methoxy-2-aza-2-butene) is thus deduced to be 233 kJ mol^−1^. Why is this enthalpy of demethoxylation so much larger than the previously noted quantities?

Alternatively, let us consider the alicyclic 1-pyrroline (1-azacyclopentene) and its 2-methoxy derivative. A nearly constant value of 73 kJ mol^−1^ was found for three experimentally derived examples of the liquid-phase *N*-methylamide/*O*-methylimidate enthalpy difference [37]. We will assume that this difference is seemingly essentially independent of the amide and the corresponding imidate. The enthalpy of formation of *N*-methylpyrrolidone is approximately −268 kJ mol^−1^ [42,43], and so the enthalpy of formation of liquid 2-methoxy-1-pyrroline is deduced to be −195 kJ mol^−1^. The enthalpy of formation of gaseous 1-pyrroline was experimentally determined to be 63 kJ mol^−1^ [39]. The enthalpy of liquefaction of pyrroline was taken as that of pyridine with a total of four carbons [41], namely 25 kJ mol^−1^. The enthalpy of formation of liquid 1-pyrroline is thus derived as 38 kJ mol^−1^; thus, the demethoxylation enthalpy of liquid 2-methoxy-1-pyrroline is 233 kJ mol^−1^, the same as before.

Finally, let us consider 1-piperideine (1-azacyclohexene) and its 2-methoxy derivative. We previously said that the liquid-phase *N*-methylamide/*O*-methylimidate enthalpy difference, 73 kJ mol^−1^, is seemingly essentially independent of the amide and the corresponding imidate. Let us choose this consensus value, which in fact is indistinguishable from the measured value [37]. The measured enthalpy of formation of *N*-methyl-2-piperidone is −293 kJ mol^−1^ [44], and so the enthalpy of formation of liquid 2-methoxy-1-piperideine is deduced to be −220 kJ mol^−1^. The quantum-chemically calculated enthalpy of formation of 40 kJ mol^−1^ for gas-phase 1-piperideine [39] and its estimated enthalpy of vaporization, set equal to that of pyridine itself (41 kJ mol^−1^ from [41]) result in enthalpy of formation for liquid 1-piperideine of 1 kJ mol^−1^. The demethoxylation enthalpy of 2-methoxy-1-piperideine is deduced to be −221 kJ mol^−1^, which is in good accord with our earlier result.

The conclusions regarding the plausibility of the data are summarized in Table 2 containing the demethoxylation enthalpies and the formation enthalpies of the corresponding pairs of compounds (methoxylated and not) (Table 2). All details such as the formula and phase of the compound, method of measurement or derivation, reference citations, and corresponding thermochemical information about a few other related nonalkaloidal nitrogen-containing species are to be found in the body of this paper. What numerical regularities are there for demethoxylation enthalpies?

(a) Demethoxylation of acyclic and alicyclic species with the -C(OCH_3_)=N- functionality, such as *N*,*O*-dimethylacetimidate (3-methoxy-2-aza-2-butene), 2-methoxypiperidine, and 2-methoxypyrroline, is accompanied by a ~230 kJ mol^−1^ increase to more positive enthalpies of formation.
(b) Demethoxylation of heterocyclic species with the -C(OCH_3_)=N- functionality, such as 2-methoxypyridine and 2,6-dimethoxypyridine, is accompanied by a ~200 kJ mol^−1^ increase to more positive enthalpies of formation.
(c) Demethoxylation of heterocycles containing nitrogen but with the methoxy group not on the carbon adjacent to nitrogen, such as anisine, brucine, cinchonidine, papaverine, 4-methoxypyridine, 6-methoxyquinoline, and quinine, is accompanied by a ~170 kJ mol^−1^ increase to more positive enthalpies of formation.

Some quantitative understanding, or at least greater appreciation, of these three classes of demethoxylation enthalpies, can be gained from a consideration of that for isoelectronically related oxygen species in which O^+^ formally replaces N. (Admittedly, in what follows, only gas-phase values are discussed here because the necessary measured data for condensed phase species are absent). In the particular, consider now protonated methyl *p*-methoxybenzoate, [*p*-CH_3_OC_6_H_4_C(OH)OCH_3_]^+^. In a formal sense, this species may be demethoxylated by a process related to a) to form protonated *p*-methoxybenzaldehyde, [*p*-CH_3_OC_6_H_4_CHOH]^+^. Likewise, we may simulate process c) by the alternative demethoxylation to form protonated methyl benzoate, [C_6_H_5_C(OH)OCH_3_]^+^.

Which process is less endothermic? Because [*p*-CH_3_OC_6_H_4_C(OH)OCH_3_]^+^ is shared by the two demethoxylation reactions, it suffices to look at only the difference of the enthalpies of formation of the isomeric ions [*p*-CH_3_OC_6_H_4_CHOH]^+^ and [C_6_H_5_C(OH)OCH_3_]^+^. In turn, we can derive each of these quantities from knowledge of the enthalpies of formation of the appropriate carbonyl compound (and of H^+^), and the negative (by definition) of the proton affinity. Ignoring now the shared value of the enthalpy of formation of H^+^, taking the enthalpies of formation of *p*-anisaldehyde and methyl benzoate [45,46] and both proton affinities from the archival review [47], we find [*p*-CH_3_OC_6_H_4_CHOH]^+^ is less stable than [C_6_H_5_C(OH)OCH_3_]^+^ by nearly 70 kJ mol^−1^. Accordingly, the enthalpies of the two demethoxylation reactions for *p*-CH_3_OC_6_H_4_C(OH)OCH_3_]^+^ that form [*p*-CH_3_OC_6_H_4_CHOH]^+^ and [C_6_H_5_C(OH)OCH_3_]^+^ differ by this 70 kJ mol^−1^. This difference is numerically very similar to the 230 − 170 = 60 kJ mol^−1^ difference for earlier processes (a) and (c) employed in the understanding of alkaloids and related nitrogen-containing species.

## 3. Conclusions

In the present paper, we discuss the wide, diverse and sophisticated chemistry of alkaloids as befits their general complexity as (poly)cyclic compounds with many nitrogens and multiple functional groups and therefore low volatility. There is almost a total absence of knowledge of the energetics of these species as manifest by their enthalpy of formation, a fortiori in the thermochemically idealized gas phase. To remedy this lack of knowledge of vaporization and sublimation data alike, we considered the “plausibility” of the few literature referenced numbers for condensed-phase alkaloids. This plausibility was most generally achieved through the evaluation of demethoxylation enthalpies of alkaloids, and related simpler nitrogen-containing species.

## Figures and Tables

**Table 1:** Trivial name, molecular formula, CASRN, and IUPAC name of the selected compounds.

	Methoxylated Species	Demethoxylated Species
Trivial name	quinine (**1**)	cinchonidine (**2**)
Molecular formula	C_20_H_24_N_2_O_2_	C_19_H_22_N_2_O
CASRN	130-95-0	485-71-2
IUPAC name ^1^	(*R*)-(6-methoxyquinolin-4-yl)((1*S*,2*S*,4*S*,5*R*)-5-vinylquinuclidin-2-yl)methanol	(*R*)-quinolin-4-yl((1*S*,2*S*,4*S*,5*R*)-5-vinylquinuclidin-2-yl)methanol
Trivial name	quinidine (**3**)	cinchonine (**4**)
Molecular formula	C_20_H_24_N_2_O_2_	C_19_H_22_N_2_O
CASRN	56-54-2	118-10-5
IUPAC name ^1^	(*S*)-(6-methoxyquinolin-4-yl)((1*S*,2*R*,4*S*,5*R*)-5-vinylquinuclidin-2-yl)methanol	(*S*)-quinolin-4-yl((1*S*,2*R*,4*S*,5*R*)-5-vinylquinuclidin-2-yl)methanol
Trivial name	brucine (**5**)	strychnine (**6**)
Molecular formula	C_23_H_26_N_2_O_4_	C_21_H_22_N_2_O_2_
CASRN	357-57-3	57-24-9
IUPAC Name ^1^	(4*aR*,5*aS*,8*aR*,13*aS*,15*aS*,15*bR*)-10,11-dimethoxy-4*a*,5,5*a*,7,8,13*a*,15,15*a*,15*b*,16-decahydro-2*H*-4,6-methanoindolo [3,2,1-ij]oxepino[2,3,4-de]pyrrolo[2,3-h]quinolin-14-one	(4*aR*,5*aS*,8*aR*,13*aS*,15*aS*,15*bR*)-4*a*,5,5*a*,7,8,13*a*,15,15*a*,15*b*,16-decahydro-2*H*-4,6-methanoindolo[3,2,1-ij]oxepino[2,3,4-de]pyrrolo[2,3-h]quinolin-14-one
Trivial name	anisine (**7**)	amarine (**8**)
Molecular formula	C_24_H_24_N_2_O_3_	C_21_H_18_N_2_
CASRN	575-55-1	91044-33-6
IUPAC name ^1^	(4*R*,5*S*)-2,4,5-tris(4-methoxyphenyl)-4,5-dihydro-1*H*-imidazole	(4*R*,5*S*)-2,4,5-triphenyl-4,5-dihydro-1*H*-imidazole
Trivial name	papaverine (**9**)	- (**10**)
Molecular formula	C_20_H_21_NO_4_	C_16_H_13_N
CASRN	58-74-2	6907-59-1
IUPAC name ^1^	1-(3,4-dimethoxybenzyl)-6,7-dimethoxyisoquinoline	1-benzylisoquinoline

^1^ As generated by ChemDraw Ultra 12.0 Software (CambridgeSoft Corporation).

**Table 2:** Condensed phase enthalpies of formation of methoxylated and demethoxylated species; alkaloids (all necessarily solid) and related species.

Methoxylated Species ^1^	Δ*H*_f_^o^ (kJ mol^−1^)	Demethoxylated Species	Δ*H*_f_^o^ (kJ mol^−1^)
Quinine (*n* = 1)	−160	Cinchonidine	30
Quinidine (*n* = 1)	−155	Cinchonine	31
Brucine (*n* = 2)	−496	Strychnine	−172
Anisine (*n* = 3)	−264	Amarine	213
Papaverine (*n* = 4)	−503	1-Benzylisoquinoline	150
2,4-Dimethoxypyrimidine (*n* = 2)	−277	Pyrimidine	133
2,4,6-Trimethoxy-1,3,5-triazine (*n* = 3)	−479	1,3,5-Triazine	172
6-Methoxyquinoline (*n* = 1)	−21	Quinoline	141
2-Methoxy-1-pyrroline (*n* = 1)	−195	1-Pyrroline	38
*N*,*O*-Dimethylacetimide (*n* = 1)	−204	2-Aza-2-butene	29

^1^ *n* = number of methoxy groups.

## Data Availability

Not Applicable.
